# A case study in statistical software development for advanced evidence synthesis: the combined value of analysts and research software engineers

**DOI:** 10.1186/s12874-024-02450-9

**Published:** 2025-01-17

**Authors:** Naomi Bradbury, Tom Morris, Clareece Nevill, Janion Nevill, Ryan Field, Suzanne Freeman, Nicola Cooper, Alex Sutton

**Affiliations:** 1 Biostatistics Research Group, Department of Population Health Sciences, University of Leicester, Leicester, UK; 2 Complex Reviews Synthesis Unit (CRSU), Universities of Leicester and Glasgow, Glasgow, UK; 3 Health Economics and Health Technology Assessment (HEHTA), School of Health and Wellbeing, University of Glasgow, Glasgow, UK

**Keywords:** Academic scientific software development, Web applications, Apps, Evidence synthesis

## Abstract

**Background:**

Since 2015, the Complex Reviews Synthesis Unit (CRSU) has developed a suite of web-based applications (apps) that conduct complex evidence synthesis meta-analyses through point-and-click interfaces. This has been achieved in the R programming language by combining existing R packages that conduct meta-analysis with the *shiny* web-application package. The CRSU apps have evolved from two short-term student projects into a suite of eight apps that are used for more than 3,000 h per month.

**Aim:**

Here, we present our experience of developing production grade web-apps from the point-of-view of individuals trained primarily as statisticians rather than software developers in the hopes of encouraging and inspiring other groups to develop valuable open-source statistical software whilst also learning from our experiences.

**Key challenges:**

We discuss how we have addressed challenges to research software development such as responding to feedback from our real-world users to improve the CRSU apps, the implementation of software engineering principles into our app development process and gaining recognition for non-traditional research work within the academic environment.

**Future developments:**

The CRSU continues to seek funding opportunities both to maintain and further develop our *shiny* apps. We aim to increase our user base by implementing new features within the apps and building links with other groups developing complementary evidence synthesis tools.

**Supplementary Information:**

The online version contains supplementary material available at 10.1186/s12874-024-02450-9.

## Background

The Complex Reviews Synthesis Unit (CRSU) is a collaboration between the Universities of Leicester and Glasgow that has existed since 2015 (previously known as the Complex Reviews Support Unit until April 2023). During this time, an aim of the group has been to support systematic reviews and other related projects for the Cochrane collaboration and other NIHR and NHS funded programmes.

A systematic review of medical interventions for a given disease often includes a statistical analysis, in the form of one or more meta-analyses (MAs). When several interventions are evaluated, each pair of interventions can be compared in a pairwise meta-analysis. Alternatively, or in addition, a single network meta-analysis (NMA) can be used to compare all the interventions within the same model. NMAs are more difficult to carry out than traditional pairwise MAs, and typically require programming within specialist statistical software [1–3].

Another common form of MA is of studies of diagnostic test accuracy (DTA) [4]. These differ from ordinary pairwise MAs in that each study contributes two correlated outcomes, the diagnostic test’s sensitivity and specificity. As a result, DTA-MAs are also more challenging to conduct than standard pairwise MA.

Over the past nine years, the CRSU has sought to make these and other advanced MA methods accessible to non-specialists through the development of eight interactive web applications [5]. Each app uses a graphical point-and-click interface that does not require software to be installed beyond a web browser. These apps can be gathered under three broad headings.

1. Apps to conduct evidence synthesis of treatment effects. These are:

- **MetaPairwise** for standard pairwise meta-analysis [6].
- **MetaImpact** allows the user to calculate the power of a theoretical new study to affect the result of a pairwise meta-analysis once included [7].
- **MetaInsight** for NMAs with Normal or Binomial outcomes [8, 9].
- **MetaCNMA** is for component-NMA, in which there are several basic interventions, or *components*, various combinations of which comprise the full interventions [10].

2. Apps to conduct evidence synthesis of diagnostic test accuracy studies.

- **MetaDTA** allows DTA-MAs to be carried out under the frequentist framework [11–13].
- **MetaBayesDTA** allows DTA-MAs to be carried out under the Bayesian framework and has further features such as imperfect gold standard latent class models [14, 15].

3. Other apps that do not fit under the above two groups.

- **DTA-Primer** is an interactive tutorial on the basics of diagnostic test-accuracy.
- **MetaInsightCOVID** is a modified version of MetaInsight focusing on pharmacological interventions for COVID-19 that was developed as part of a feasibility study for interactive reporting of (living) NMAs during the height of the pandemic [16].

All the CRSU apps were created in the R programming language using the *shiny* package that allows R code to be run via an interactive app [17]. These can be deployed to the internet as web apps, bypassing the need for more traditional web development languages such as ASP.NET and PHP. All apps are packaged with example datasets so that the new user can explore their full functionality. In addition, apart from DTA-Primer and MetaInsightCOVID, each app includes data upload functionality, allowing users to conduct their own meta-analysis and create data visualisations that can be viewed on screen or downloaded to be included in reports or publications.

The CRSU apps use existing R packages where possible to conduct the meta-analyses and create data visualisations. For example, *netmeta* and *gemtc* are among the packages used for frequentist and Bayesian network meta-analysis respectively [18, 19]. Likewise, *lme4* is used to fit the frequentist statistical models in MetaDTA while *RStan* provides the functionality to fit the Bayesian statistical models used in MetaBayesDTA and MetaCNMA [3, 20]. By leveraging existing, well-regarded R packages, the team at CRSU were able to focus their efforts on developing the novel, interactive aspects of the apps.

**Fig. 1:**
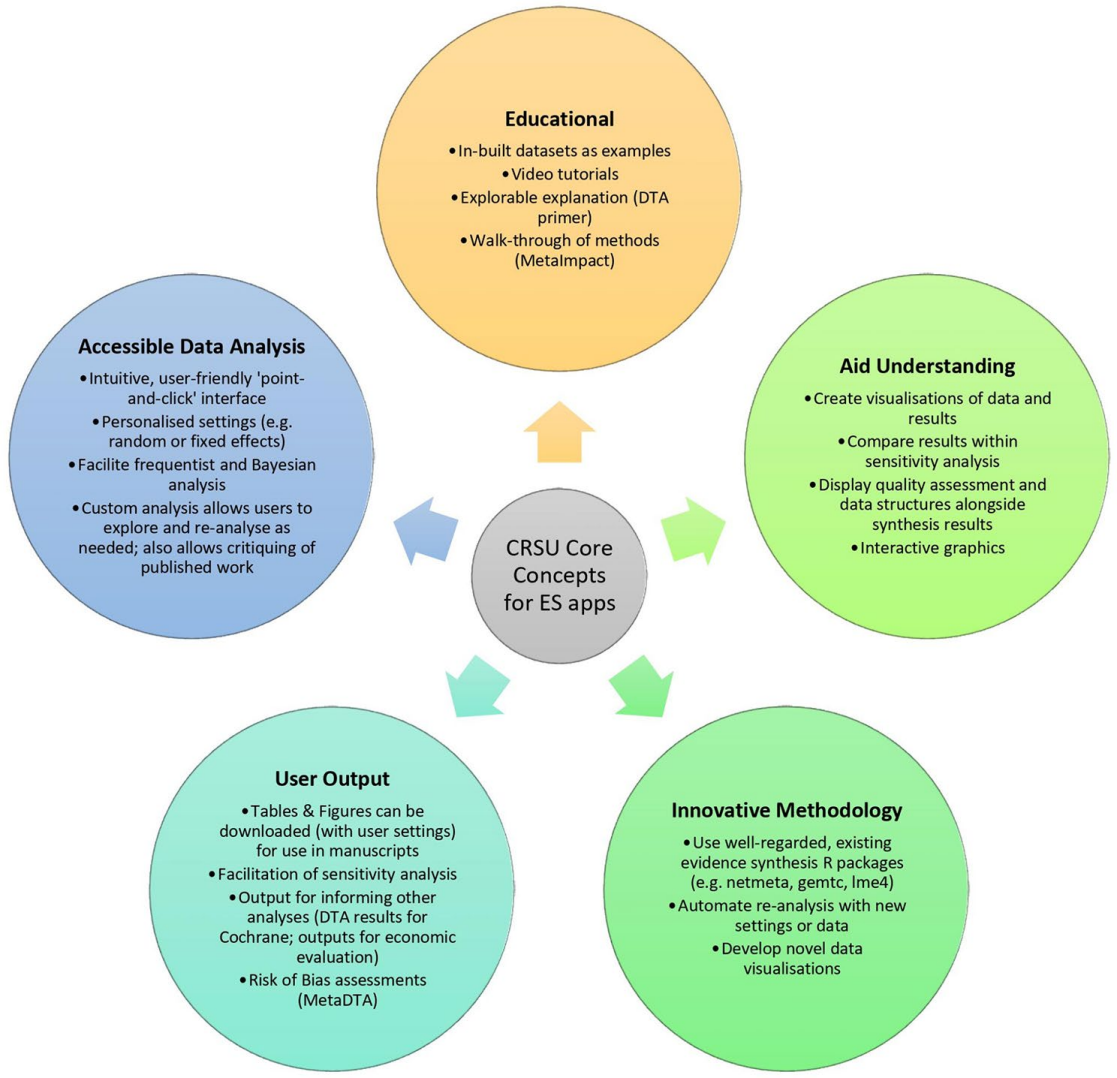
Innovating Evidence Synthesis Technology: the CRSU experience. The core concepts of the CRSU approach that users will find integrated into our software

The original aim of the CRSU project was to develop user-friendly interfaces to enable non-technical researchers to utilise complex evidence synthesis methods. Figure 1 provides an overview of how this original project aim was superseded and the remit expanded, with each of these topics being discussed within this paper. We keep these core concepts in mind when developing all our software to give consistency and deliver high-impact outputs through innovative and accessible data analysis and visualisation that enhances users’ understanding of advanced evidence synthesis methods. All code for our apps is licensed under the GNU General Public Licence that ensures our software remains free to use and also allows users to edit the source code within the terms of the licence [21]. Initially our apps were licensed using GitHub’s more restrictive default licence as we were not aware of the open-source licensing available, but the team now embraces this more open and fairer way of developing code [22].

**Fig. 2:**
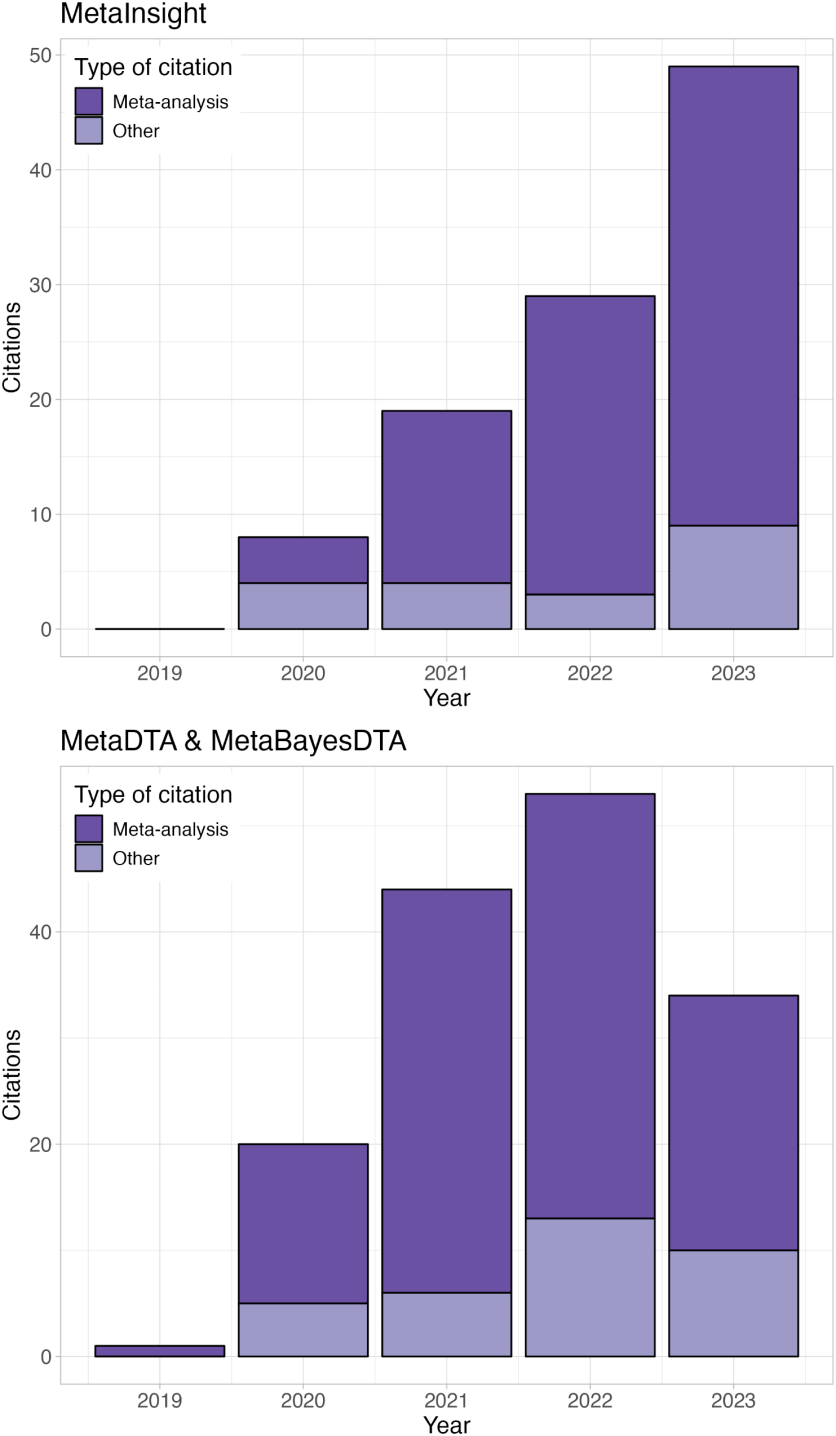
Forward citations of CRSU publications. Total number of citations by year for MetaInsight (top) [9, 24] and DTA apps (MetaDTA and MetaBayesDTA combined) (bottom) [12, 13, 15] from publication in 2019 until 15th November 2023; darker shade represents papers that have specifically used the apps to conduct their own meta-analysis

As of the end of 2023, the CRSU apps have collectively been cited in over 250 research publications (Fig. 2**)** as well as in the Cochrane Handbook for Systematic Reviews of Diagnostic Test Accuracy ( [23], Chap. 10, page 10) and the 2021 European Stroke Organisation and European Academy of Neurology joint guidelines on post-stroke cognitive impairment. They have also been demonstrated in various training workshops, from which user feedback has been very positive. Appendix 1 contains detailed information on how the citation searching process was conducted to produce Fig. 2.

### Pre-history and tentative beginnings

Interest in the use of NMA grew quickly in the early years of the new millennium. From a UK perspective, this coincided with the formation of the National Institute for Health and Care Excellence (NICE). NICE commenced conducting technology appraisals to decide whether emerging technologies were effective and cost-effective compared to all competing comparators and, therefore, should be recommended in England and Wales for routine use in the National Health Service. The need for the estimation of the relative comparative effectiveness of multiple interventions for NICE appraisals provided added impetus to existing research groups developing NMA methodology and led to the quick acceptance and uptake of this methodology by the research community.

NICE were early adopters of NMA and their use was quickly recommended in their Methods Guide [25]. At the time, only code in specialist Bayesian software packages, such as WinBUGS, was available to conduct NMA [26]. This led to a shortage of expertise to conduct such analyses.

**Fig. 3:**
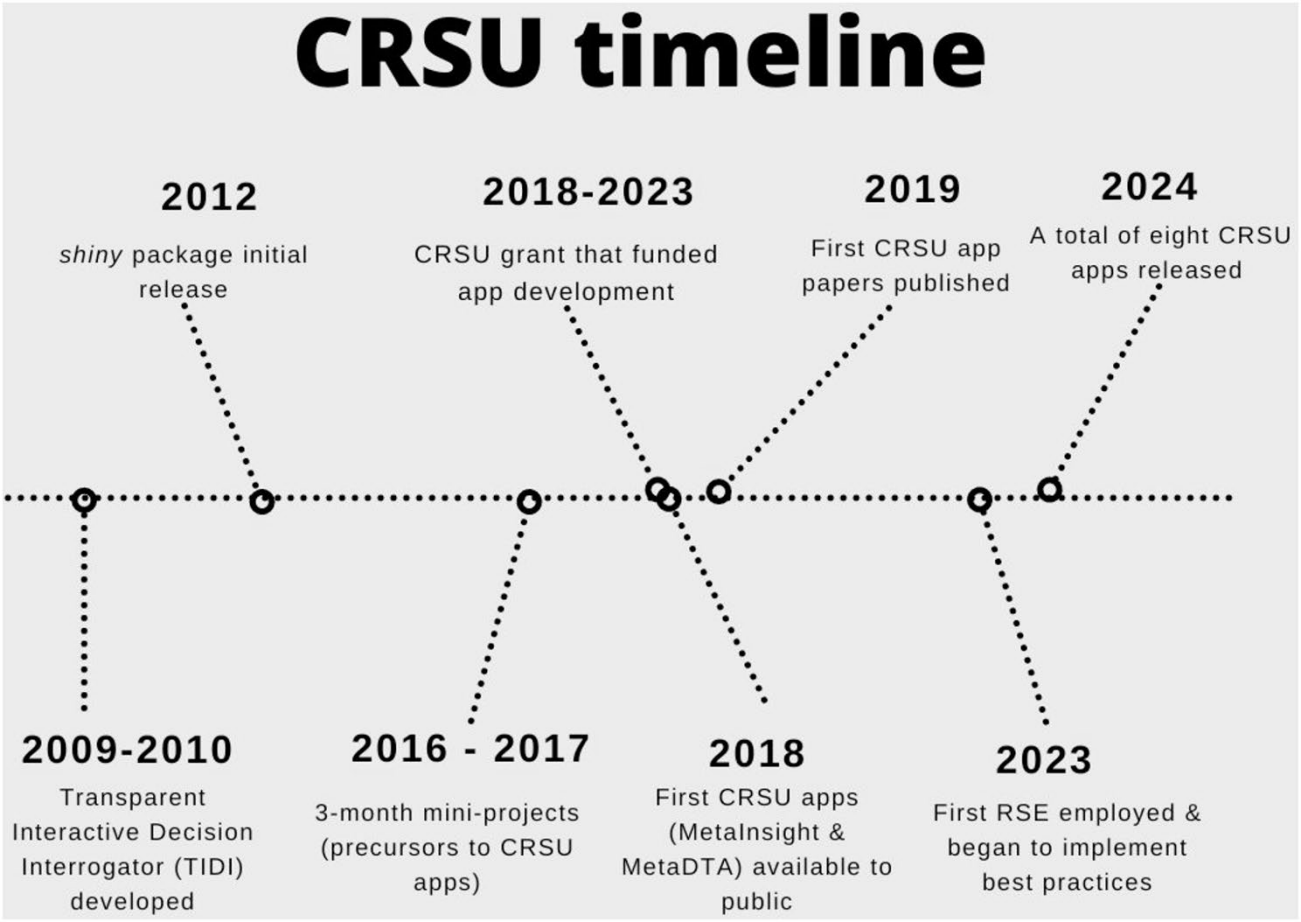
A narrative timeline of the CRSU project

Two of the authors of this paper (NC & AS) were involved both with NMA methods development and NICE appraisals at this time and observed first-hand the need and desirability of more user-friendly software. Further, these authors were involved in an ambitious feasibility project which cumulated in the development of software to modify the parameters of a NICE decision model, including the meta-analyses which informed its parameters, in real-time in an appraisal committee meeting [27]. This well-received work gave the decision makers the option of critiquing the analysis by exploring the impact of alternative assumptions. The system developed was called TIDI, which stood for Transparent Interactive Decision Interrogator. TIDI was created using a combination of R, Excel and WinBUGS linking the latter with R via existing linking packages (Fig. 3**)**. Whilst this project was a success, the software was difficult to install successfully on different PCs and required bespoke programming for each app and, thus, had fundamental limitations. However, TIDI was at the boundary of what could be achieved without specialised software engineering knowledge.

When *shiny* for R was released a short number of years later, it became clear that this package could greatly facilitate and advance many of the aims of the TIDI prototype (Fig. 3). Two of the authors (NB & CN) conducted 3-month student projects investigating the feasibility of using *shiny* to create apps to conduct advanced evidence syntheses analyses (Fig. 3). Both produced encouraging results, but the student authors moved on and, for a short time, the apps were not developed further.

Shortly after the CRSU was formed, it was identified that software was still a limiting factor for the uptake of appropriately complex evidence synthesis models for our client base. We decided that the timeliest approach to resolving this unmet software need was to do further work on the aforementioned apps that subsequently evolved into the first versions of MetaDTA and MetaInsight (Fig. 3).

Following the publication of initial papers describing each app in 2019, and presentations and workshops for the CRSU client base, both apps were well received by the research community. Since this time both apps have been periodically updated, and numerous further related app developments were initiated as described in the remainder of this paper.

### MetaInsightCOVID

The year 2020 is often known for being the beginning of the COVID-19 (coronavirus disease 2019) pandemic. By 19th October 2020, there were more than 40.1 million confirmed cases of COVID-19 worldwide and 1.1 million associated deaths [28]. Attempts to find treatments and vaccines led to an explosion of COVID-19 related research. By 5th October 2020, there had been 2,388 clinical trials registered worldwide looking into treating COVID-19 [29]. Furthermore, by 19th October 2020, 338 systematic review protocols for treatment of COVID-19 had been registered [30].

The constant influx of studies, the fact that so little was known about COVID-19, and the importance of having up-to-date knowledge created the optimal situation for conducting a living systematic review (LSR); an extension to standard review methods where researchers continuously search for new evidence and incorporate said evidence as soon as it is published [31]. During 2020, several groups worldwide had started creating LSRs on the topic of COVID-19, with associated websites that were frequently updated with the newest evidence (The COVID-NMA Initiative [32], EPPI Center [33], and The LIVING Project [34]). However, in the first year of the pandemic, none of these were utilising NMA.

As CRSU already had an established web-app for conducting and automating NMA, and motivated to improve COVID-19 research methods, we created a tailored version of MetaInsight, namely MetaInsightCOVID [16]. MetaInsightCOVID had all the same functionality of MetaInsight but with two additions. Firstly, the app presented data from one of the already existing COVID-19 LSR projects (The COVID-NMA Initiative [32]). Secondly, a new front page was created to summarise the latest results and present highlights of the NMA. MetaInsightCOVID therefore became a place where users could explore the data and interrogate analyses by choosing different options, with the app automatically updating to the user’s specification.

The development of MetaInsightCOVID acted as a feasibility study for automating and reporting living NMAs. As such, we updated the dataset once a week between 19th May and 19th October 2020. The data was contained in a Google spreadsheet linked directly to the app. In this way, our team only needed to add further trials to the Google spreadsheet, and the app and all its analyses would automatically update.

The COVID-19 trial data was being extracted from images on the COVID-NMA Initiative website. However, the volume of data was being updated at an ever-increasing rate as the weeks passed. Not only was new data being added, but existing data was also being updated and expanded. Since the CRSU team were manually extracting data from images, this task was becoming more time consuming each week.

One of the authors (JN), at the time an independent software engineer, offered their time to assist with the data-collection aspect of the project. They developed a Python script which downloaded all the images, compared them to the previous images, then created a new set of images, with any changes highlighted. These highlighted images were then emailed to the CRSU statistician (CN) alongside a summary of how many images had been added or updated. This allowed for the statistician to know exactly which data had been updated, without needing to manually check the entire dataset every week, drastically reducing the time commitment.

This Python script was configured to run on a Raspberry Pi computer in the early hours, every Monday morning, so that the email was in the statistician’s inbox when they began work. In an ideal situation, this script would have been able to extract the data and update the Google spreadsheet automatically, but the images were not sufficiently uniform, and the AI boom, making robust character recognition software easily obtainable and useable, had not yet begun.

Midway through the CRSU app development journey, this project highlighted both strengths and weaknesses in our app development process. Firstly, it iterated how powerful implementing interactivity into research continues to be. MetaInsightCOVID is a notable example of how sharing interactive results gives so much more to stakeholders than static versions. But secondly, the project highlighted how impactful an interdisciplinary team can be. Having a software engineer as part of the team for the first time brought a plethora of skills that we previously didn’t have and showed the potential benefits such skills would bring to developing web-apps effectively and efficiently.

### Growing a scientific software team

#### When apps get real-world users

In a typical scientific research environment, the publication of a journal article often signals the end of a research project (or at least the conclusion of that section of a project). However, like others before us, we soon came to discover that this was very different when producing and publishing scientific software [35].

In 2019, the CRSU published their first journal articles describing two web-based tools for analysing, interrogating and visualising network meta-analysis (MetaInsight [9]) and diagnostic test accuracy meta-analyses (MetaDTA [12]). As a result of these publications and other dissemination work, the apps began to get real-world users. This was excellent news for the success of the apps but having worldwide users, many beyond the client remit of the CRSU grant, also required the CRSU to provide user support. This could be time consuming and is not the kind of work that is typically recognised in a research grant. As the CRSU apps allow the users to upload their own data for analysis, a common problem was that users’ data were not formatted in the correct way. This produced errors on screen prompting the users to contact the app developers. We created documentation to provide guidance and troubleshooting for the most common problems. As some users wish to upload confidential data to the apps or conduct particularly computationally expensive analyses that are slow to run through our servers, we provide guidance on how to download the code so the apps can be run on the users’ own machines. More recently, we have implemented changes that allow much more flexibility in how users can upload their data, including more user-friendly upload features and utilising the newly available *rio* package [36].

**Fig. 4:**
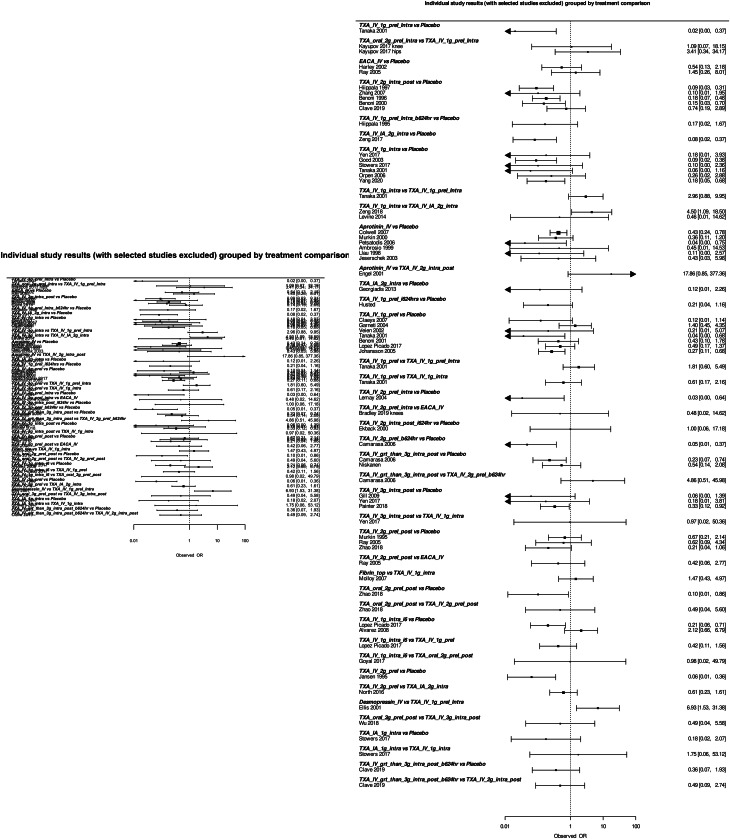
Making changes based on user experience. An example of display issues with a data visualisation for a dataset containing many studies (left) and following coding changes to create dynamically sized plots (right)

User queries were also of benefit to the development of the apps. For example, large datasets resulted in display problems with some of the data visualisations. This prompted improvements that dynamically changed the rendered size of the graphical display based on study size (Fig. 4). In addition, users contacted us with requests for additional features that they wanted to see in the apps. This meant that new developments could be focused on areas where there was high demand as well as providing evidence of the importance of these new developments to funding bodies.

#### Bringing software engineering to bear

For a long time, the coding of the CRSU apps was conducted entirely by health statisticians and epidemiologists with an interest in developing apps using *shiny* for R, rather than those with a background in software development. In addition, apps were typically worked on by one researcher at a time before being handed on to another solo developer. It became clear that this was not the optimal way of working. It became difficult to hand-on development of apps to new developers as the codebase was not optimally structured.

In 2022, the CRSU began to recruit members to the team with more varied backgrounds. Just as it was essential to have qualified health statisticians as part of the team to provide statistical expertise, it was also vital to have individuals with software engineering experience who could contribute to improving software development practices. Several members of the team also sit somewhere in between these areas, having knowledge and experience of both software development and statistical techniques; these members fall under the definition of research software engineers (RSE) [37].

This team has made large changes in the development of the CRSU software to implement software development best practices [38–40]. This includes:

- Using Git through GitHub for version control and group development of code [41, 42]. For example, the main branch on GitHub now represents the version of the code for each app that is currently deployed with development branches being used for new features. This allows multiple developers to be working on a single app at the same time.
- Use of project boards such as Jira to track features and user feedback.
- Code review of new code to improve consistency and quality of code.
- Modularisation of code to break the codebase into smaller chunks making it easier to maintain and reuse.
- Implementing automated testing of the code.
- Implementing style guidelines for code and documentation.
- Regular software developer meetings to agree coding standards and guidelines. These decisions are then documented to ensure that all developers are working to the same minimum standard. This also allows new developers to more easily be integrated into the team.

These changes have been made with the aim of improving practices as we go forward which should ensure the apps can be maintained and improved into the future.

### Lessons learned

#### Prototypes become production & packages get updated

**Fig. 5:**
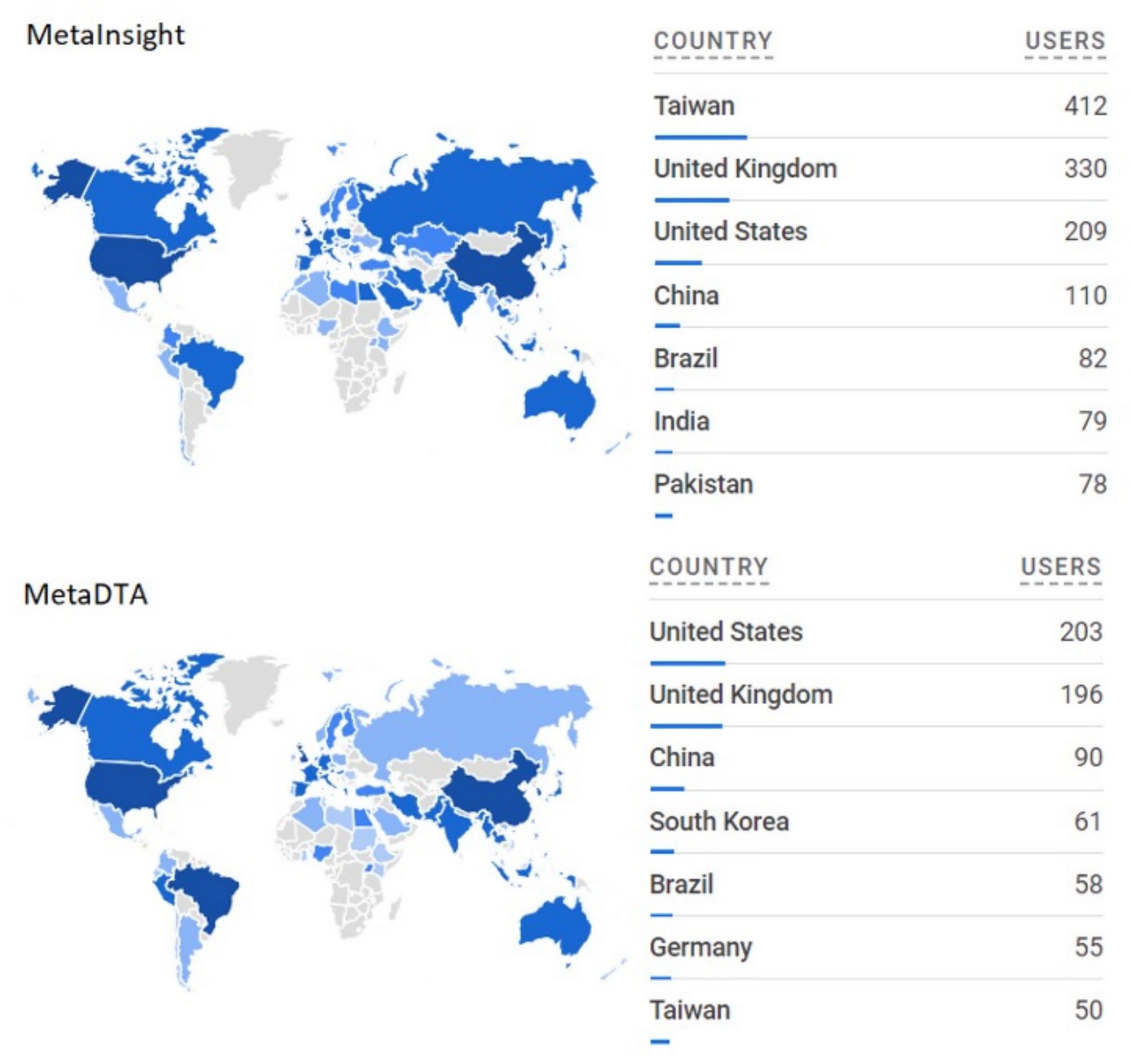
Google analytics data. Google Analytics of app users by country during 01 Apr – 28 Nov 2023 for MetaInsight and MetaDTA

When MetaInsight and MetaDTA started as 3-month student projects back in 2017, the team did not envision that these apps would go on to be used by hundreds of researchers around the world (Fig. 5**)**. To ensure the short projects were completed in time, some features within the apps were implemented in a way that was functional but not necessarily optimal or robust. When development was continued by other members of the team, some of these elements were not improved, often for several years, which was not the intention of the original developers. One such example is the way treatment labels were initially loaded into MetaInsight. A quick way to implement this feature was to have users input the treatment labels into a text box, but that led to lots of quirks with the way the text had to be formatted which resulted in many user queries. It was not until 2023 that the data entry for MetaInsight was updated, allowing users to directly include the treatment labels in the uploaded .csv file. Furthermore, some of the R packages used behind the scenes are still in active development and features that were not initially available became possible later (for example, being able to rotate labels in network plots with *netmeta* was a later addition). Therefore, it is important to stay up-to-date and regularly revisit packages for new features that may be applicable.

These experiences can be summarised into the following advice: (i) be aware that prototypes often become production, and (ii) avoid being of the mindset of ‘it works, so I don’t need to touch it’. If the assumption that these apps would become a global resource was made back in 2017, features and workflows would have been prioritised differently.

#### Balancing developing new features vs. maintenance in an academic environment

One of the eternal struggles with software development is striking a balance between new functionality and maintenance. In academia, this seems to be even more pronounced because progress is often measured through publishing academic journal papers [43]. Traditional journals are unlikely to accept a paper on maintenance even if it is a big overhaul of an entire system. It’s just not of interest to their readers. The Society of Research Software Engineering is making progress on this, by showing academic institutions the value in this kind of work [44]. Furthermore, there are other organisations which are building new ways of recognising software contributions to research, such as The Journal of Open Source Software and the Software Sustainability Institute [45, 46]. From the outset of a project, it is important to plan for the time and funds that will need to be devoted to software maintenance.

#### Software engineers are an important part of a scientific software development team

Since conception, we have always emphasised the importance of statistical support to users of our apps. Our apps can facilitate the processes of conducting complex statistical analyses, but statistical support is key to ensuring the correct complex statistical analyses are undertaken in the first place and the results are interpreted correctly. In hindsight, the same principle could and should be applied to software development.

The profession of research software engineer and their role within academic scientific software is relatively new and we did not get an RSE involved in our apps until 2023 [47]. However, involving a software engineer from the beginning would allow strong foundations to be built in a way that lends itself to future enhancements, even if those enhancements are currently unknown, and thus avoid the need for ‘workarounds’ which remain in the apps for many years. Furthermore, as interactive apps increase in popularity, peer reviews of journal article submissions are asking about best practices such as testing and the FAIR principles [48, 49]. Involvement from an RSE from the beginning can help ensure that best practices become routine from the start rather than something to be adopted later.

### Future developments

#### Funding

Funding ongoing development and maintenance of research software is an ongoing challenge for groups developing open source software within the university sector [43]. One member of our team contributed to a recent report into the difficulties of funding open source scientific software [50]. However, we continue to ensure we have resources to maintain the apps so that they remain available to our users. From 2018 until 2023, the development of the CRSU apps was funded entirely through an NIHR grant. We have recently been successful in gaining funding for app maintenance and further development through the Chan Zuckerberg Initiative (CZI) Essential Open Source Software for Science programme (Cycle 6) being funded through the Wellcome Trust [51].

Web app hosting inevitably has costs associated with it. Therefore, there is a need to ensure a plan is in place so the apps can continue to be available into the future, accounting for potential increases in usage and, thus, cost. Alternative methods of hosting are being piloted including self-hosting via university infrastructure.

When applying for funding to continue the development of existing apps, it is beneficial to be able to provide quantifiable measures of usage. For example, if hosting *shiny* apps on shinyapps.io servers, basic usage statistics are provided through the shinyapps.io website [52]. Analytics tools such as Google Analytics can be embedded within *shiny* apps and provide much greater detail on where in the world your app users are located (Fig. 5) as well allowing for many other reporting features [53]. Publishing journal articles detailing the novel features of an app helps reach new users as well as providing a more traditional measure of success for funding bodies.

#### User experience

We are also aware of many ways in which the user experience of the apps can be improved as well as additional functionality that we would like to incorporate. As mentioned above, it was not until 2023 that we had a software engineer directly involved with our projects and we started to introduce standard software engineering principles including best practices and automated testing. As such, some of the older codebases would benefit from being refactored to improve on the code quality. As we have not yet had specific funding for code maintenance, we have made improvements to the codebase alongside development work where possible. For example, a major update to MetaInsight in July 2024 (v6.0.0) included new functionality (meta-regression) as well as a lot of ‘behind the scenes’ work to implement *shiny* modules and unit testing [54].

Over the years of the CRSU project, developments in *shiny* have made it easier to create apps with more modern user interfaces and some of our more established apps would benefit from interface improvements to make the user experience more consistent across the suite of apps. Accessibility of the apps is also important and therefore, whilst we have introduced colour-blind friendly palette options on some graphical presentations, the apps would benefit from further accessibility testing. Many of our users use the visual and statistical outputs from our apps in scientific publications. We would like to improve the level of customisation offered by our plots and tables to make it even easier for users to download publication quality data visualisations directly from the apps.

From a user perspective, the apps are designed to be point-and-click with the underlying analysis code hidden from the user. However, this lack of reproducible analysis is a barrier to the potential uptake of the apps by organisations such as NICE and Clinical Trial Units. The ability to view the analytical code would improve both transparency and reproducibility and will assist all users in conforming with open science standards. Further benefits include: (1) novice users can communicate exactly the analyses they have done by showing experienced analysts their code; (2) If an app does not do exactly what the user wants, but comes close (e.g. minor modifications to a figure are required), a user could conduct the vast majority of work with the app but seamlessly swap to the R programming language when desired; and (3) Seeing the underlying code could have educational value for a novice user seeking to improve their skills in using the lower-level package. This feature is currently being actively explored.

As developments continue in the field of Medical Statistics and statisticians adopt ever increasingly complex analysis techniques, we receive requests from users to implement additional functionality within our apps. At the time of writing, we have just completed extending the MetaInsight app to incorporate meta-regression, including baseline risk adjustments. In addition, MetaCNMA (set to be released imminently), will allow users to conduct component network meta-analysis.

Finally, while the CRSU apps have been shown to be used globally and the outputs presented in scientific publications, there is still a large potential userbase who are unaware the apps exist. We have run workshops at academic conferences and observed sustained increased use of the apps as a result. We will continue to offer these where possible and target multiple groups including academia and industry.

### The future of statistical analysis software

The development of packages such as *shiny* has made it easier for teams that do not think of themselves as traditional software developers, to create interactive ways of communicating their research. The ways in which academic research is disseminated to stakeholders is still based predominantly on text and static visuals despite the huge increases in computational capability.

The use of interactive reporting, be that through apps, interactive dashboards or other dynamic formats furthers the objectives of Open Science to make scientific research available to all members of society. Integrating data, analysis and reporting into one workflow makes it quicker and easier for the analyst to update and explore the data as well as creating a format that encourages transparency of the scientific process. The interactive format is engaging and allows the reader the freedom to go beyond passive consumption, perhaps gleaning additional insights beyond those of the original authors.

Although there are challenges with developing scientific software within a research environment, we hope this insight into our experience as the CRSU will provide encouragement for others to follow in this innovative area of research.

## Electronic supplementary material

Below is the link to the electronic supplementary material.

Supplementary Material 1

## Data Availability

No datasets were generated or analysed during the current study.

## Abbreviations

CZI: Chan Zuckerberg Initiative
COVID-19: Coronavirus Disease 2019
CRSU: Complex Reviews Synthesis Unit (formerly Complex Reviews Support Unit)
DTA: Diagnostic Test Accuracy
ES: Evidence Synthesis
MA: Meta-Analysis
NICE: National Institute for Health and Care Excellence
NMA: Network Meta-Analysis
RSE: Research Software Engineer
TIDI: Transparent Interactive Decision Interrogator
