# Supplementary material for: A case study in statistical software development for advanced evidence synthesis: the combined value of analysts and research software engineers
Source: BMC Med Res Methodol. 2025 Jan 17;25:13. doi: 10.1186/s12874-024-02450-9 (PMC11740572; doi:10.1186/s12874-024-02450-9)
Supplement: Supplementary file 1 — Supplementary Material 1 [file 12874_2024_2450_MOESM1_ESM.docx]

**Appendix 1 –** Citation searching process to produce **Figure 2**

**Methods**

The five CRSU papers published by November 2023 were included in the forward citation search. These five papers were placed into two groups:

- Network meta-analysis papers relating to the development of MetaInsight (Owen 2019 and Nevill 2023) [1, 2].
- Diagnosic Test Accuracy papers on the development of MetaDTA (Freeman 2019, Patel 2021) and MetaBayesDTA (Cerullo 2023) [3-5].

The forward citations were extracted using the citationchaser app on 15^th^ November 2023 [6]. The resulting RIS files were downloaded from citationchaser and then converted to xml files in EndNote. At this stage, where citations of the papers included within that forward citation search had been incorporated they were manually removed.

The resulting xml files were uploaded to the SR-Accelerator deduplicator tool to remove duplicate citations followed by using the SR-Accelerator for the screening process [7]. The full text of each paper was screened to place the article into one of the two following groups:

*Meta-analysis*

- Authors clearly state that they have used the relevant CRSU app for some or all of their analysis or data visualisations
- When this was not clearly defined, articles were included where the figures appeared to be produced from the CRSU apps

*Other*

- CRSU app cited but not directly used for analysis i.e. overview of methods, papers describing other Shiny apps, book chapter
- Authors desired to use a CRSU app but their data was not sufficient
- Protocols
- Not journal articles (conference proceedings, letters etc)
- No full-text or English text access

Where errors were discovered at this stage (duplications, retracted papers or papers that were not forward citing) they were removed from the analysis.

**Table A1 – Forward citation numbers at each step of the citation searching process**

| **Year** | **MetaInsight**  (Owen 2019 & Nevill 2023) | **DTA apps**  (Freeman 2019, Patel 2021 and Cerullo 2023) |
| --- | --- | --- |
| citationchaser | 115 citations, 113 unique | 217 citations, 174 unique |
| xml file | 111 | 172 |
| Deduplicated | 106 | 164 |
| **Screenatron/Final** | 105 | 152 |

**Table A2 - MetaInsight results** – number of forward citations of MetaInsight grouped by year from 2019 to 2023

| **Year** | **Total citations** | **Citation that used MetaInsight to conduct NMA (Meta-analysis group)** |
| --- | --- | --- |
| 2019 | 0 | 0 |
| 2020 | 8 | 4 |
| 2021 | 19 | 15 |
| 2022 | 29 | 26 |
| 2023 (until 15^th^ Nov) | 49 | 40 |
| **Total** | **105** | **85** |

**Table A3 - MetaInsight results** – number of forward citations of DTA apps grouped by year from 2019 to 2023

| **Year** | **Total citations** | **Citation that used apps to conduct DTA-MA** |
| --- | --- | --- |
| 2019 | 1 | 1 |
| 2020 | 20 | 15 |
| 2021 | 44 | 38 |
| 2022 | 53 | 40 |
| 2023 (until 15^th^ Nov) | 34 | 24 |
| **Total** | **152** | **118** |

**References**

1. Owen, R.K., et al., *MetaInsight: An interactive web-based tool for analyzing, interrogating, and visualizing network meta-analyses using R-shiny and netmeta.* Res Synth Methods, 2019. **10**(4): p. 569-581.

2. Nevill, C.R., N.J. Cooper, and A.J. Sutton, *A multifaceted graphical display, including treatment ranking, was developed to aid interpretation of network meta-analysis.* J Clin Epidemiol, 2023. **157**: p. 83-91.

3. Freeman, S.C., et al., *Development of an interactive web-based tool to conduct and interrogate meta-analysis of diagnostic test accuracy studies: MetaDTA.* BMC Medical Research Methodology, 2019. **19**(1): p. 81.

4. Patel, A., et al., *Graphical enhancements to summary receiver operating characteristic plots to facilitate the analysis and reporting of meta-analysis of diagnostic test accuracy data.* Res Synth Methods, 2021. **12**(1): p. 34-44.

5. Cerullo, E., et al., *MetaBayesDTA: codeless Bayesian meta-analysis of test accuracy, with or without a gold standard.* BMC Medical Research Methodology, 2023. **23**(1): p. 127.

6. *citationchaser.* <https://estech.shinyapps.io/citationchaser/>.

7. Clark, J., et al., *A full systematic review was completed in 2 weeks using automation tools: a case study.* J Clin Epidemiol, 2020. **121**: p. 81-90.
